# Hidden Aggregation Hot-Spots on Human Apolipoprotein E: A Structural Study

**DOI:** 10.3390/ijms20092274

**Published:** 2019-05-08

**Authors:** Paraskevi L. Tsiolaki, Aikaterini D. Katsafana, Fotis A. Baltoumas, Nikolaos N. Louros, Vassiliki A. Iconomidou

**Affiliations:** Section of Cell Biology and Biophysics, Department of Biology, National and Kapodistrian University of Athens, Panepistimiopolis, Athens 15701, Greece; etsiolaki@biol.uoa.gr (P.L.T.); k.katsafana@gmail.com (A.D.K.); fbaltoumas@biol.uoa.gr (F.A.B.); nlouros@biol.uoa.gr (N.N.L.)

**Keywords:** apolipoprotein E, amyloid fibrils, Alzheimer’s disease, Αβ oligomer

## Abstract

Human apolipoprotein E (apoE) is a major component of lipoprotein particles, and under physiological conditions, is involved in plasma cholesterol transport. Human apolipoprotein E found in three isoforms (E2; E3; E4) is a member of a family of apolipoproteins that under pathological conditions are detected in extracellular amyloid depositions in several amyloidoses. Interestingly, the lipid-free apoE form has been shown to be co-localized with the amyloidogenic Aβ peptide in amyloid plaques in Alzheimer’s disease, whereas in particular, the apoE4 isoform is a crucial risk factor for late-onset Alzheimer’s disease. Evidence at the experimental level proves that apoE self-assembles into amyloid fibrilsin vitro, although the misfolding mechanism has not been clarified yet. Here, we explored the mechanistic insights of apoE misfolding by testing short apoE stretches predicted as amyloidogenic determinants by AMYLPRED, and we computationally investigated the dynamics of apoE and an apoE–Αβ complex. Our in vitro biophysical results prove that apoE peptide–analogues may act as the driving force needed to trigger apoE aggregation and are supported by the computational apoE outcome. Additional computational work concerning the apoE–Αβ complex also designates apoE amyloidogenic regions as important binding sites for oligomeric Αβ; taking an important step forward in the field of Alzheimer’s anti-aggregation drug development.

## 1. Introduction

Human mature apolipoprotein E (apoE) is a 299 amino acid glycoprotein [1,2], taking part in most lipoprotein classes, such as chylomicrons, very low-density lipoproteins (VLDL) and high-density lipoproteins (HDL) [3]. It is a member of an apolipoprotein family, along with apoA-I, apoA-II, apoA-IV, ApoC-I, apoC-II, and apoC-III [4,5]. Each apolipoprotein class has distinct functions and participates actively in the formation of specific lipoprotein scaffolds [6]. Human mature apolipoprotein E is primarily synthesized in the liver, where it is found in higher quantities, but it is also a protein of the brain and other tissues [7]. The functional form of the protein is involved in metabolic pathways that are related to plasma cholesterol and triglyceride transport and distribution among the tissues, by interacting with members of the low-density lipoprotein receptor (LDLR) superfamily [8,9,10,11].

The *APOE* gene [12], co-localized with the *APOC1* [12,13] and *APOC2* genes [14,15,16], has three alleles; *APOE2*, *APOE3* and *APOE*4 [17,18]. Each allele exhibits distinct frequencies among the human population, with *APOE3* having the highest (approximately 78%) [19,20,21]. The expression of these alleles results in three main forms of the protein, namely, apoE2, apoE3, and apoE4. Interestingly, the apoE4 isoform is of great importance, since it is reported to be involved in both hereditary and sporadic types of the Alzheimer’s disease (AD) [22,23,24]. The differences among the three forms are restricted in the positions 112 and 158 of the mature polypeptide chain. More specifically, in apoE2, cysteines are located in both positions, whereas in apoE4 there is an arginine in both positions. In apoE3, on the other hand, there is a cysteine in position 112 and an arginine in position 158 [25].

**Figure 1:**
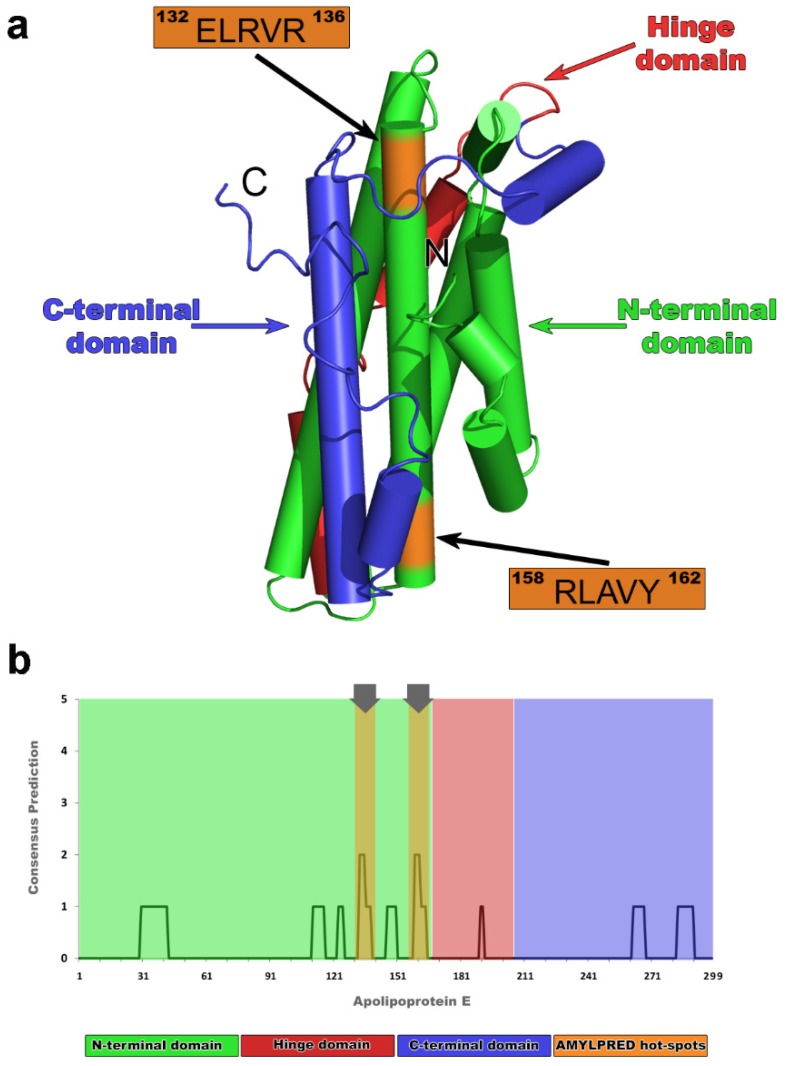
Native nuclear magnetic resonance (NMR) structure of human mature apolipoprotein E (apoE) [33] and apoE amyloidogenic profile by AMYLPRED [37]. (**a**) Different colors show all three structural domains of the apoE3 in solution: the N-terminal domain (green);the C-terminal domain (blue); and the hinge domain (red). Colored regions in orange illustrate “aggregation-prone” segments ^132^ERLVR^136^ and ^158^RLAVY^162^, respectively, both located on the 4th helix of the four-helix bundle. (**b**) Amyloid propensity apoE histogram represents a weak overall amyloidogenicity, since only two segments exceed the consensus AMYLPRED threshold (regions ^132^ERLVR^136^ and ^158^RLAVY^162^). Color scheme follows the rules described in (**a**).

Apolipoprotein E is found in both lipid-bound and lipid-free forms. Lipid-free species are relatively rare and are possibly the result of transient dissociation events during the lipoprotein creation [26,27,28,29,30,31]. It has not been yet possible for any lipid-free form of apoE to be crystallized in the monomeric form, due to its tendency to assemble in tetramers or octamers [32]. A nuclear magnetic resonance (NMR) structure, with the addition of several mutations, successfully determined the three-dimensional conformation of an apoE lipid-free monomer [33]. According to the model, supported by the experimental outcome of the NMR structure, apoE has three structural domains: the N-terminal domain (Figure 1a, green), the C-terminal domain (Figure 1a, blue), and the hinge domain (Figure 1a, red). The monomer connectivity includes the association of the Ν-terminal domain (residues 1–167) [34,35] with the C-terminal domain (residues 206–299) [36] through a short interim hinge domain (residues 168–205) [33]. Part of the N-terminal domain adopts a four-helix bundle conformation, which is proposed to be the domain buried in the interior of the lipid-free particle [33] (Figure 1a, green).

Lipid-free apolipoproteins related to apoE are implicated with several amyloidosis [38] as a result of their proneness to misfold [39]. ApoE self-accumulation properties are still poorly understood, although—as mentioned above—the APOE4 allele is known as a causative risk factor for the neurodegenerative AD [40,41]. ApoE has been characterized as a potential Aβ chaperone in AD, suggesting the strong tendency between these two macromolecules to interact. Interestingly, apoE misfolding was proposed as the first step towards Αβ nucleation and polymerization. In any case, the outstanding appearance of apoE in AD and other neurodegenerative diseases is attributed to the fact that lipid transport in cerebrospinal fluid (CSF) is mediated by HDL particles rich in apoE [42,43,44].

In the context of the “amyloid stretch hypothesis”, which proposes that amyloidogenesis is actually driven by short fragments of misfolded proteins [45], scientists have extensively been studying a variety of short aggregation-prone stretches, with a potential to guide amyloid fibril formation from a soluble globular domain [46,47,48,49,50,51,52,53]. Based on this idea, many algorithms have been developed, in an attempt to extract the information of amyloidogenicity only from primary protein sequences [54]. Among them, AMYLPRED, a consensus prediction algorithm developed in our lab [37], was used to identify regions with amyloidogenic properties in the amino acid sequence of apoE (Figure 1b). The ultimate aim of the present study was to characterize the amyloidogenic properties of apoE3—the most common form in human population. For this purpose, we have used a combination of TEM, X-rays, polarizing microscopy ATR-FTIR spectroscopy, and molecular dynamics simulations to test whether the predicted apoE fragments can influence aggregation of either apoE or the oligomeric Aβ interacting partner. Our biophysical approach indicates that two aggregation-prone apoE hot-spots (Figure 1a, peptides ^132^ELRVR^136^ and ^158^RLAVY^162^ shown in orange) have strong self-association properties and destabilize the apoE lipid-free topology. Further, molecular details of the interaction between apoE and oligomeric Αβ, derived by our computational results, also profile the impact of hidden amyloidogenic apoE regions in AD.

## 2. Results and Discussion

### 2.1. Computational Identification of apoE Hot-Spots

After a computational scanning, AMYLPRED revealed a weak overall amyloidogenic tendency for apoE, in contrast to other amyloidogenic apolipoproteins studied before [55,56]. The consensus prediction recognized two regions of apoE, namely, ^133^LRV^135^ and ^159^LAV^161^, as peptides with aggregation potency that exceeds the AMYLPRED threshold (Figure 1b). Both peptides werelocated in the same α-helix corresponding to the N-terminal four-helix bundle domain (Figure 1a, orange). According to AMYLPRED, predicted aggregation hot-spots were only found in the helix bundle of apoE that includedthe primary binding epitope for both lipids and Αβ [57], although previous in vitro aggregation assays revealed the C-terminal part as the most amyloidogenic apoE domain [58]. Arginine 112, rendering ApoE4 the least stable apoE isoform [23,59], does not affect the amyloidogenic profile of different apoEs. Analogous hot-spots traced in all apoE forms since the ^133^LRV^135^ peptide is a commonly predicted segment for all three apoEs, while the ^159^LAV^161^ was found only in the apoE3 and apoE4 isoforms (Figure S1). The ^133^LRV^135^ peptide is an important functional region, since it is neighboring to the LDL receptor binding domain of the molecule [34]. It has been suggested that the C-terminal apoE domain dissociates causing exposure of the four-helix bundle of apoE [33]. This finding is in good agreement with our prediction and verifies the idea that aggregation-prone regions are not buried [37]. We hypothesize that a critical apoE conformational transition can uncover both ^133^LRV^135^ and ^159^LAV^161^ aggregation-prone segments, and thus, can initiate apoE misfolding (See MD results below). In this study, predicted regions were extended from both ends, following the idea that five-residue-long peptides are sufficient to independently form amyloid-like fibrils [60], and thus, ^132^ELRVR^136^ and ^158^RLAVY^162^ pentapeptide–analogues were experimentally used to pinpoint segments that play crucial role in the self-assembly process of apoE and in the molecular recognition of Aβ.

### 2.2. Isolated apoE Peptide–Analogues Fulfill All Basic Amyloid Criteria

**Figure 2:**
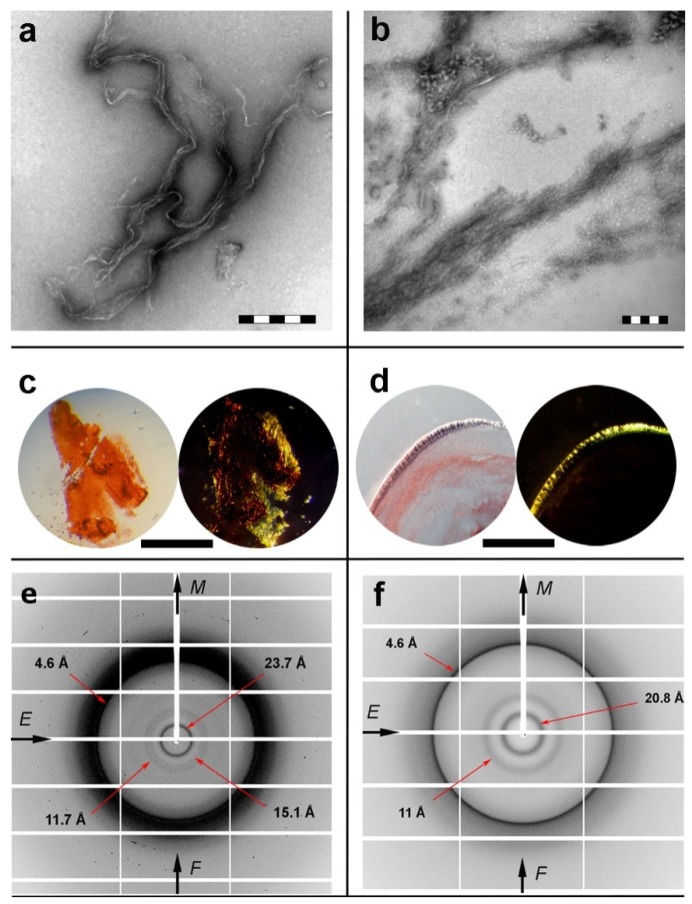
Experimental results of self-aggregation assays for apoE peptide–analogues. (**a**,**b**) Electron micrographs of typical amyloid fibrils, derived by self-assembly of (**a**) ^132^ERLVR^136^ and (**b**) ^158^RLAVY^162^ “aggregation-prone” fragments. Scalebars for (**a**) ^132^ERLVR^136^ and (**b**) ^158^RLAVY^162^ are 200 nm and 500 nm, respectively. (**c,d**) Photomicrographs of apoE peptide fibrils stained with the amyloid specific Congo red dye ((**c)** ^132^ERLVR^136^ and (**d**) ^158^RLAVY^162^). The apple-green birefringence, characteristic for all amyloid fibrillar materials, is clearly seen (Scale bar 500 μm).(**e**,**f**) X-ray diffraction patterns from oriented fibers of apoE “aggregation-prone” fragments, (**e**) ^132^ERLVR^136^ and (**f**) ^158^RLAVY^162^.

Designed apoE peptide–analogues ^132^ELRVR^136^ and ^158^RLAVY^162^ were thoroughly examined and found to self-assemble, forming fibril-containing gels after an incubation period of one week. As observed by negative staining TEM, both ^132^ERLVR^136^ and ^158^RLAVY^162^ fibrillar populations were measured to have similar diameters (Figure 2a,b). The thinnest single fibril of the ^132^ELRVR^136^ peptide–analogue hadan average diameter of 100 Å, whereas the ^158^RLAVY^162^ peptide thickness wasapproximately 110 Å. However, the overall arrangement of the fibrils in each gel seems to differ between the two peptides, possibly owing to differences between the peptide–peptide interactions, acting as building blocks of the fibrillar core [61]. Congo red was shown to selectively bind on thin hydrated films derived by both peptides, as seen under bright field illumination. The characteristic yellow/green birefringence wasclearly seen under crossed polars of a polarizing microscope (Figure 2c,d).

**Figure 3:**
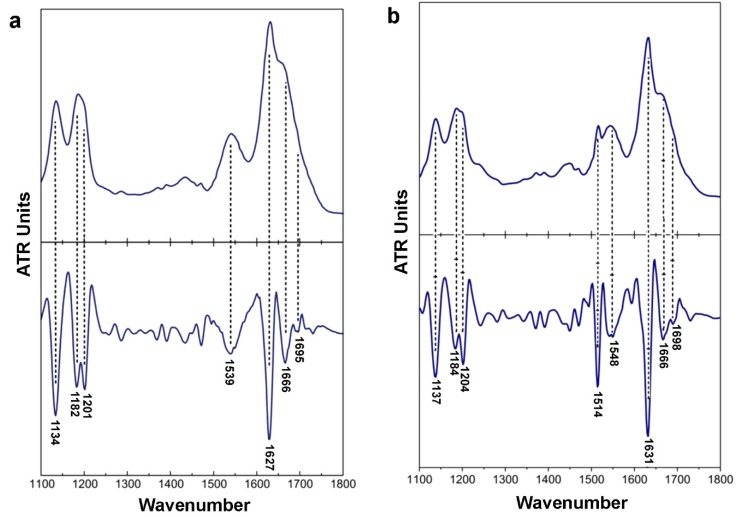
FT-IR spectra (1100–1800 cm^−1^) derived from suspensions of fibrils, produced from (**a**) ^132^ERLVR^136^ and (**b**) ^158^RLAVY^162^. Each apoE peptide cast on a flat stainless-steel plate and left to air-dry slowly at ambient conditions to form hydrated, thin films. Each film possesses a β-sheet conformation, as it is evident by the presence of strong amide I and II bands.

X-ray fiber diffraction and FT-IR experiments have all shown that in their fibrillary form both peptides adopt a well-defined β-sheet conformation. The X-ray patterns indicate that fibrils from both the ^132^ELRVR^136^ and ^158^RLAVY^162^ peptide–analogues possess the typical “cross-β” architecture of amyloid fibrils (Figure 2e,f). Concerning the ^132^ELRVR^136^, a strong -but diffuse- 4.6 Å reflection is seen in the diffraction pattern, in addition to an 11.7 Å structural repeat. The former reflections may be attributed to the periodic distance between consecutive hydrogen-bonded β*-*strands, which are aligned perpendicular to the fiber axis, and the repetitive distance between packed β-sheets aligned parallel to the fiber axis, respectively. In addition to the typical “cross-β” repetitions, a reflection measured at 23.7 Å could be indicative of the inter-sheet distance (half of the 23.7 Å is approximately 11.7 Å), indicating a long-range order of packed β-sheets in the fiber. Finally, the reflection at 15.1 Å may be attributed to the length of the extended ^132^ERLVR^136^peptide. The respective reflections in the diffraction pattern of the ^158^RLAVY^162^ peptide were measured to be at 4.6 Å, representing the repetitive interchain distance between β-strands and 11 Å, corresponding to the inter-sheet stacking periodicity, both closely resembling typical “cross-β” patterns taken from amyloid fibrils. An additional spacing at 20.8 Å is the evidence for the distance between ordered and packed β-sheets (half of the 20.8 Å is approximately 11 Å). Reflections were also verified utilizing ZipperDB [62] models that overlap with ^132^ELRVR^136^ and ^158^RLAVY^162^ peptide–analogues (data not shown). ATR FT-IR was subsequently used to access the secondary structure characteristics of both peptides and to verify the results derived by X-rays. An ATR FT-IR spectrum of a thin-film cast from suspensions of the amyloid-like fibrils of the peptide–analogue^132^ERLVR^136^ (Figure 3a) shows prominent bands at 1627 cm^-1^and 1539 cm^-1^, in the amide I and II regions, respectively, indicating the presence of *β-*sheets. A band at 1695 cm^-1^is indicative of anti-parallel *β-*sheets (Table 1). Similarly, in the spectrum of ^158^RLAVY^162^ (Figure 3b), the bands at 1631 cm^-1^ (amide I) and 1548 cm^-1^(amide II) are also attributed to *β-*sheets, whereas the band at 1689 cm^-1^ is attributed to anti-parallel *β-*sheets (Table 1).

Our experimental analysis reveals that apoE peptide–analogues ^132^ELRVR^136^ and ^158^RLAVY^162^ hada strong propensity to independently form β-aggregates, fulfilling the basic amyloid criteria. This finding is compatible with the proposed apoE aggregation pathway suggesting that a minor apoE fraction forms β-strands that stabilize the apoE fibril core [63].

### 2.3. Implication of apoE Peptide–Analogues in the Tertiary Structural Stability of apoE

Molecular dynamics simulations were carried out on the most representative NMR conformer of apoE3 [33], putting the spotlight on the implication of the experimentally tested amyloidogenic peptide–analogues ^132^ELRVR^136^ and ^158^RLAVY^162^. Computational tests assessed the structural stability, integrity, and dynamic behavior of apoE over time (300 ns) under physiological pH conditions at 300 K. Structural movements were monitored over the course of the simulations through time-dependent root mean square deviation (RMSD) measurements with respect to the starting configuration, to evaluate apoE overall structural transitions, as well as through per-residue root mean square fluctuation (RMSF) calculations to monitor the mobility of specific regions.

**Figure 4:**
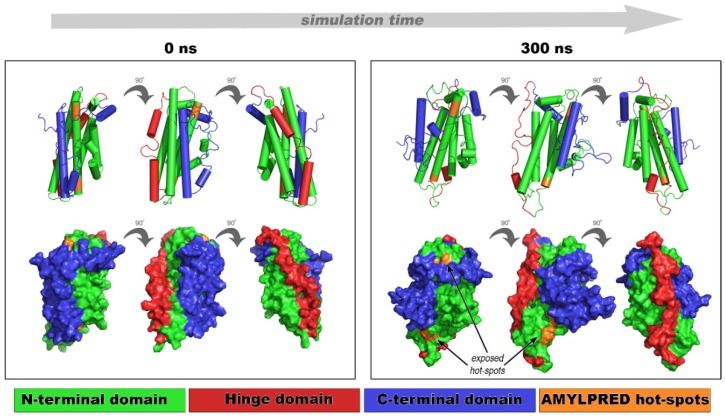
Dynamics simulations of an apoE NMR structure for 300 ns. The N-terminal domain is shown in green, the C-terminal domain is shown in blue, and the hinge domain is shown in red. “Aggregation-prone” hot-spots ^132^ERLVR^136^ and ^158^RLAVY^162^ are colored in orange. Structural movements uncover otherwise hidden apoE hot-spots (arrows). Models are represented in 0°, 90°, and 180°.

Fibril-forming segments (^132^ELRVR^136^ and ^158^RLAVY^162^) influence the apoE structural features over time, since a noticeable difference found between the starting conformation (Figure 4, 0 ns frames) and the 300 ns conformation (Figure 4, 300 ns frames). The N-terminal domain kept its bundle-structure throughout the simulation, and only a slight conformational tilt was observed in the 3D shape of the molecule (Figure 4, 300 ns frames). Conversely, the C-terminal domain was characterized by large fluctuations (8–10 Å) with respect to the N-terminal domain, possibly due to the higher solvent exposure (Figure S2a, blue curve). Root mean square fluctuation calculations reveal approximately 10 Å deviation between residues Glu270 and His299, corresponding to the C-terminal domain (Figure S3). This result is common for apoE since similar conformational changes allow the four-helix bundle to emerge during lipid binding [33]. It is also believed that C-terminal fluctuations allow new interactions or α-helix to β-sheet conversion, due to partial destabilization of apoE, subsequently resulting in self-assembling. In either case, conformational instability of the C-terminal domain exposes aggregation-prone segments ^132^ELRVR^136^ and ^158^RLAVY^162^, otherwise hidden in the core of apoE. The overall conformational variations of segments ^132^ELRVR^136^ and ^158^RLAVY^162^ are visually inspected in Figure S2. ^158^RLAVY^162^ exhibited higher conformational mobility, meaning that this segment participatedin transient C-terminal conformational changes (Figure S2b, orange triangles). The conformational unraveling of the most aggregation-prone part of apoE (according to AMYLPRED, Figure 1a) explains the intrinsic apoE propensity to form amyloid-like fibrils [63]. Our aggregation assays in combination with computational MD results suggest that the C-terminal domain protects the aggregation-prone part of apoE from misfolding, by covering the aggregation-prone regions ^132^ELRVR^136^ and ^158^RLAVY^162^ located at the N-terminal domain. This finding is in agreement with the computational analysis by Das and Gursky [55].

### 2.4. An apoE Aggregation Hot-Spot Anchors Oligomeric Aβ

Numerous studies demonstrate that apoE is a component of peripheral deposits and senile plaques of AD patients [64,65,66]. In vitro experiments have shown that co-incubation of apoE3 and apoE4 with Αβ peptide induces the fibrillation of the peptide [67,68], supporting the idea that apoE specifically interacts with the Aβ. One mechanism by which apoE might be involved in the pathology of AD is by modulating the activity of Aβ and binding in oligomeric Aβ species [69,70]. Molecular docking was employed towards the identification of the Αβ and apoE epitopes in the Aβ–apoE complex. The Aβ aggregation profile was analyzed utilizing AMYLPRED (Figure S4). Two aggregation-prone regions were predicted comprising an N-terminal pentapeptide (KLVFFA) and a longer C-terminal thirteen-residue-long peptide (GAIIGLMVGGVVI) (Figure S4). Previous experimental studies have shown that both regions have self-aggregation properties and have been suggested as crucial amyloidogenic determinants of Αβ [71].

Supervised molecular docking was performed for building the Aβ–apoE complex using the 300 ns apoE conformation as the initial structure for the N-terminal apoE domain (described above) and the 2BEG NMR structure [72] as the 3D structure of Aβ oligomers. The apoE binding epitope was restricted between residues 130 to 165, based on reliable information from experimental and computational studies, pinpointing this apoE domain as the major interacting part of apoE withΑβ [57]. Predicted Aβ aggregation-prone regions were used as computational restraints in HADDOCK. The identification of the complex ascertained the interaction between the C-terminal aggregation-prone epitope of Aβ and the amyloidogenic ^132^ELRVR^136^ peptide, located at the N-terminal apoE domain. This cluster evaluated having the best HADDOCK score, whichcorresponds to the smallest weighted HADDOCK sum (Figure S5, 0 ns).

Having investigated the most favorable Aβ–apoE complex, the next step was to evaluate its dynamics and stability. After 100 ns all-atom MD simulations, a complex dissociation was observed and the structure of the Αβ oligomer changed. Similar results were observed after 200 and 300 ns simulation time (Figure S5). Despite the secondary structure alterations, the interaction interface between Aβ and apoE retained over time. This verifies recursively the spatial position emerged from the molecular docking model (Figure S5, 0 ns). The structure of the apoE N-terminal domain was recorded as the most stable entity, since this domain displayed similar dynamic behavior over time. This behavior is consistent with the simulation results observed for the representative NMR conformer of full-length apoE3, presented above (Please refer to Section 2.3). Except for significant changes in Αβ orientation and stability, the Αβ C-terminal epitope remained constantly attached to the amyloidogenic ^132^ELRVR^136^ peptide over ns time. The Αβ oligomer’s instability wasreasonable, since oligomeric states are significantly unstable compared to the amyloid state of proteins [73]. Given the competitive relationship between lipid-free apoE molecules tending to self-assemble, and Aβ oligomers “willing” to interact with apoE monomers [74,75], we hypothesized that these computational results give new insights into Aβ–apoE’s delicate interconnection. This computational outcome provides some details into the intermolecular and intramolecular interactions, associated with the formation of homomeric or heteromeric supramolecular assemblies, which may be the key to target protein misfolding diseases.

## 3. Materials and Methods

### 3.1. Identification of Aggregation-Prone Peptides in apoE and Αβ

Human apoE sequence (UniProtKB: P02649/APOE_HUMAN), corresponding to APOE3 allele, and human Amyloid-beta precursor protein (APP) fragment 672–713 (UniProtKB: P05067/A4_HUMAN), namely, Aβ_1–42_, were analyzed with AMYLPRED [37] for identifying fibril-forming aggregation hot-spots. Fibril-forming segments chosen for this study were predicted at least by two predictors (default AMYLPRED threshold). Figure S1 and Figure S4 illustrate the consensus AMYLPRED prediction for all apoE isoforms and Aβ, respectively.

### 3.2. Peptide Design, Synthesis,and Preparation of Peptide Samples

Based on the amyloidogenic profile of apoE (Figure S1), 2 short pentapeptide–analogues were designed. Since, according to previous studies, sequence stretches in proteins should comprise a minimum of five consecutive residues, and AMYLPRED predictions were extended from both ends. The pentapeptide–analogues ^132^ELRVR^136^ and ^158^RLAVY^162^, corresponding to the 4th helix of the four-helix bundle of apoE (Figure 1, orange), were chemically synthesized in high peptide purity (>98%) by GeneCust© Europe, Luxemburg. Peptide-analogues have free N- and C-terminals. Lyophilized aliquots of both pentapeptides were re-suspended in distilled water (pH 5.5) at concentrations up to 15 mg ml^-1^ and incubated at ambient temperatures for 1–2 weeks. Both pentapeptides were found to produce fibril-containing gels.

### 3.3. X-ray Diffraction

For each peptide–analogue a droplet (~10μL) of mature fibril suspension was placed between two quartz capillaries covered with wax. Capillaries spaced ~1.5 mm apart and mounted horizontally on a glass substrate, as collinearly as possible, in order to obtain an oriented fiber. The X-ray diffraction pattern from this fiber was collected at a P14 beamline synchrotron (Petra III, EMBL-Hamburg, Germany) operated at a wavelength of 1.23953 Å, with a 6M PILATUS detector. The specimen-to-film distance was set at 225.11 mm and the exposure time was set to 1 s. The X-ray patterns were initially viewed using the program CrysAlisPro [76,77] and subsequently displayed and measured with the aid of the iMosFLM [78] program [78].

### 3.4. Negative Staining and Transmission Electron Microscopy

For negative staining, droplets (3–5 μL) of the mature fibril suspensions were applied to glow-discharged 400-mesh carbon-coated copper grids for 2 min. The grids were stained with a droplet (5 μL) of 2% (*w*/*v*) aqueous uranyl acetate for 60 s and the excess staining was removed by blotting with a filter paper. The fibril-containing grids were initially air-dried and subsequently examined with a Morgagni™ 268 transmission electron microscope, operated at 80 kV. Digital acquisitions were performed with an 11-Mpixel side-mounted Morada CCD camera (Soft Imaging System, Muenster, Germany).

### 3.5. Attenuated Total Reflectance Fourier-Transform Infrared Spectroscopy (ATR FTIR) and Post-Run Computations of the Spectra

A 10-μL droplet of each apoE peptide mature fibril suspension was cast on flat stainless-steel plates, coated with an ultrathin hydrophobic layer (SpectRIM, Tienta Sciences, Inc. Indianapolis, IN, USA) and left to dry slowly at ambient conditions in order to form thin hydrated films. Infrared spectra were obtained from these films at a resolution of 4 cm^−1^, utilizing an IR microscope (IRScope II by Bruker Optics) equipped with a Ge attenuated total reflectance (ATR) objective lens (20×) and attached to a Fourier-transform infrared (FTIR) spectrometer (Equinox 55, by Bruker Optics). Ten 32-scan spectra were collected from each sample and averaged to improve the sound/noise (S/N) ratio. Both are shown in the absorption mode after correction for the wavelength dependence of the penetration depth (pd~λ). Absorption band maxima were determined from the minima in the second derivative of the corresponding spectra. Derivatives were computed analytically using routines of the Bruker OPUS/OS2 software, including smoothing over a ±13 cm^−1^range around each data point, performed by the Savitzky–Golay algorithm [79]. Smoothing over narrower ranges resulted in a deterioration of the S/N ratio and did not increase the number of minima that could be determined with confidence.

### 3.6. Congo Red Staining and Polarized Light Microscopy

Fibril suspensions of the peptide solutions were applied to glass slides and stained with a 10 mM Congo red (Sigma) solution in distilled water (pH 5.5) for ~30 min. Excess staining was removed by several washes with distilled water and left to dry for approximately 10 min. The samples were observed under bright field illumination and between crossed polars, using a Leica MZ_75_ polarizing stereomicroscope, equipped with a JVC GC-X3E camera.

### 3.7. Molecular Docking and Molecular Dynamics Simulations

For deriving a structural model of the Αβ–apoE complex, the web server version 2.2 of HADDOCK was used [80]. The HADDOCK score was used to rank and evaluate the generated clusters. The scoring wasthe weighted sum of a linear combination of various energies and buried surface area between molecules constituting the complex. A number of molecular dynamics (MDs) simulations were designed and performed for apoE next, both in its monomeric form and in its complex with Aβ protofibrils. Each protein system was inserted into a cubic solvent box, with a minimum distance of at least 1.5 nm between the box’s boundaries and protein coordinates. The solvent was modeled using the TIP3P water model [81] and the systems were ionized using NaCl counter-ions to neutralize unwanted charges and an ambient NaCl ion concentration of 0.15 M, mimicking neutral pH conditions. Each simulation system was subjected to thorough energy minimization, followed by two stages of equilibration simulations with position restraints applied on protein coordinates, namely, a 500 ps simulation in the canonical (NVT) ensemble to equilibrate temperature and a 1 ns simulation in the isothermal-isobaric (NPT) ensemble to equilibrate pressure. An additional 1 ns equilibration simulation was also performed without any restraints. Finally, production simulations were performed in the NPT ensemble for 300 ns.

All simulations were performed using GROMACS v. 2016.3 [82] and the AMBER 99SB-ILDN force field [83]. The LINCS algorithm [84] was applied to model bond constraints, enabling the use of a 2 fs time-step. Short range non-bonded interactions were modeled using a twin-range cutoff at 0.8 nm, while long-range electrostatic interactions were modeled using the Particle Mesh Ewald (PME) method [85], with a Fourier grid spacing at 0.12 nm and a cubic interpolation (PME rank 4). Temperature was maintained at 300 K with separate couplings for the proteins and solvent, using the Berendsen weak coupling algorithm [86] during equilibration and the Nosé–Hoover thermostat [87,88] in the production simulations, with a coupling constant of τ_T_ = 0.1 ps. Pressure was isotopically controlled at 1.013 bar (1 atm), using the Berendsen weak coupling algorithm [86] during equilibration and the Parrinello–Rahman barostat [89] in the production simulations, with a coupling constant of τ_P_ = 2.0 ps and a compressibility of 4.5 × 10^−5^ bar^−1^. Simulation results were analyzed using various GROMACS utilities, and Visual Molecular Dynamics (VMD) v. 1.9.4 [90]. Pictures were collected with PyMOL [91].

## 4. Conclusions

The purpose of this study was to investigate the poorly explored amyloidogenic properties of human apolipoprotein E [58,63], a protein closely associated with disorders with worldwide prevalence, such as Alzheimer’s disease [23,24]. Wild-type or mutated apolipoproteins, evolutionarily related to apoE, have been found in depositions of amyloid fibrils in vivo in several amyloidosis [92,93,94,95,96,97,98,99,100,101]. More specifically, apoCII and apoCIII has been reported to form amyloid fibrils both in vitro [102,103] and recently in vivo, causing rare forms of hereditary systemic amyloidosis [104,105]. These two newly identified fibril proteins expand the list of amyloidogenic apolipoproteins associated with amyloidoses [38] and draw attention to unknown aggregation properties of the apolipoprotein family.

In this work, AMYPRED was used in order to probe hidden amyloidogenic motifs on the apoE polypeptide chain, whereas several biophysical and computational techniques were applied to characterize its properties. The results of our experimental work prove that predicted aggregation-prone apoE peptides self-assemble into amyloid-like fibrillar structures, displaying the main structural and tinctorial features of amyloids [106,107]. Computational tests evaluated the contribution of these peptides into the stability of apoE and explored their affinity to oligomeric Αβ. Molecular dynamic simulations revealed that both predicted apoE peptide–analogues undergo a critical structural transition that under the right in vitro conditions may result in apoE instability. Importantly, the amyloidogenic ^132^ELRVR^136^ peptide, a commonly predicted segment for all three apoE isoforms, emerged as the most favorable apoE epitope “attracting”the C-terminal epitope of the oligomeric Αβ. Overall, self-aggregation properties of apoE peptides, described here, add considerable further details as they establish a mechanistic explanation of apoE misfolding and involvement with oligomeric Αβ. As an extension to these conclusions, the concept of interacting amyloidogenic regions, by separate partners found in amyloidoses, offers hope of new anti-aggregation treatment directions.

## Figures and Tables

**Table 1:** Bands observed in the ATR FT-IR spectra obtained from thin films, containing suspensions of fibrils, produced by apoE peptide–analogues, and their tentative assignments.

Wavenumber (cm^−1^)		Assignment
^132^ERLVR^136^	^158^RLAVY^162^	
1134	1137	TFA
1182	1184	TFA
1201	1201	TFA
-	1514	Tyrosine
1539	1548	β-sheet (Amide II)
1627	1631	β-sheet (Amide I)
1666	1666	TFA
1695	1689	Antiparallel β-sheets

## Supplementary Materials

Supplementary materials can be found at https://www.mdpi.com/1422-0067/20/9/2274/s1.

## Abbreviations

AD	Alzheimer’s Disease
APP	Amyloid-Beta Precursor Protein
apoE	Apolipoprotein E
CSF	Cerebrospinal Fluid
HDL	High-Density Lipoproteins
LDLR	Low-Density Lipoprotein Receptor
MD	Molecular Dynamics
NMR	Nuclear Magnetic Resonance
RMSD	Root Mean Square Deviation
RMSF	Root Mean Square Fluctuation
TEM	Transmission Electron Microscopy
VLDL	Very Low-Density Lipoproteins
