# Supplementary material for: Hidden Aggregation Hot-Spots on Human Apolipoprotein E: A Structural Study
Source: Int J Mol Sci. 2019 May 8;20(9):2274. doi: 10.3390/ijms20092274 (PMC6539603; doi:10.3390/ijms20092274)
Supplement: Supplementary file 1 [file ijms-20-02274-s001.pdf]

Supplementary file

Hidden Aggregation Hot-Spots on Human Apolipoprotein E: a Structural Study

Paraskevi L. Tsiolaki[#], Aikaterini D. Katsafana[#], Fotis A. Baltoumas, Nikolaos N. Louros, Vassiliki A. Iconomidou*

Section of Cell Biology and Biophysics, Department of Biology, National and Kapodistrian University of Athens, Panepistimiopolis, Athens, 15701, Greece

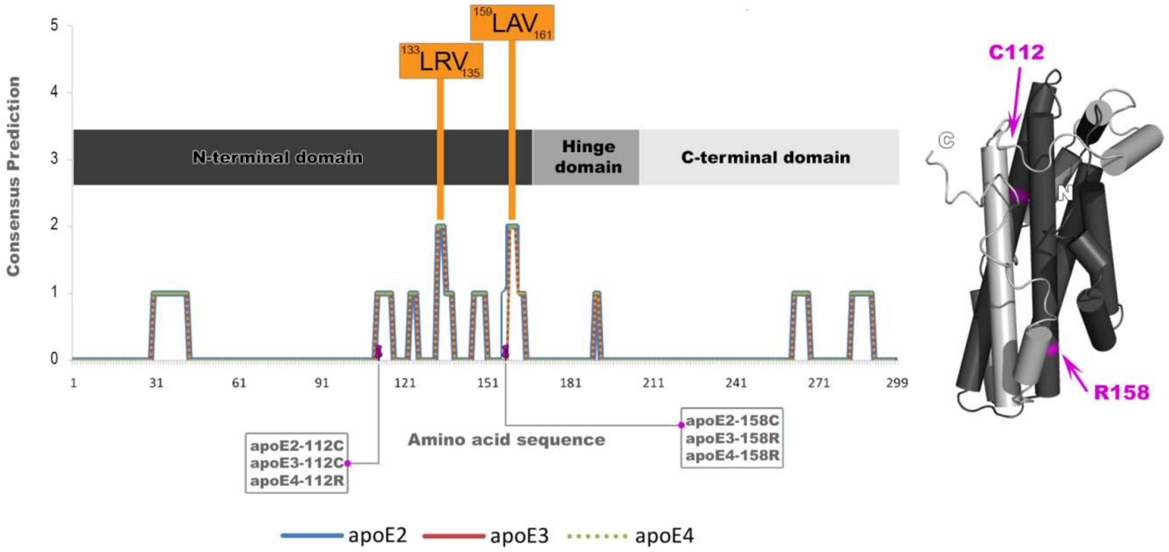

Figure S1. Amyloid propensity histograms of apoE2, apoE3 and apoE4, based on AMYLPRED [1]. Two apoE regions, namely ^{133}LRV^{135} and ^{159}LAV^{161}, were recognized as “aggregation-prone” segments (orange boxes). ApoE2 (blue line) has a slightly different profile in comparison to two other isoforms. Residues that differ between isoforms are coloured in magenta on the3D - NMR structure of apoE.

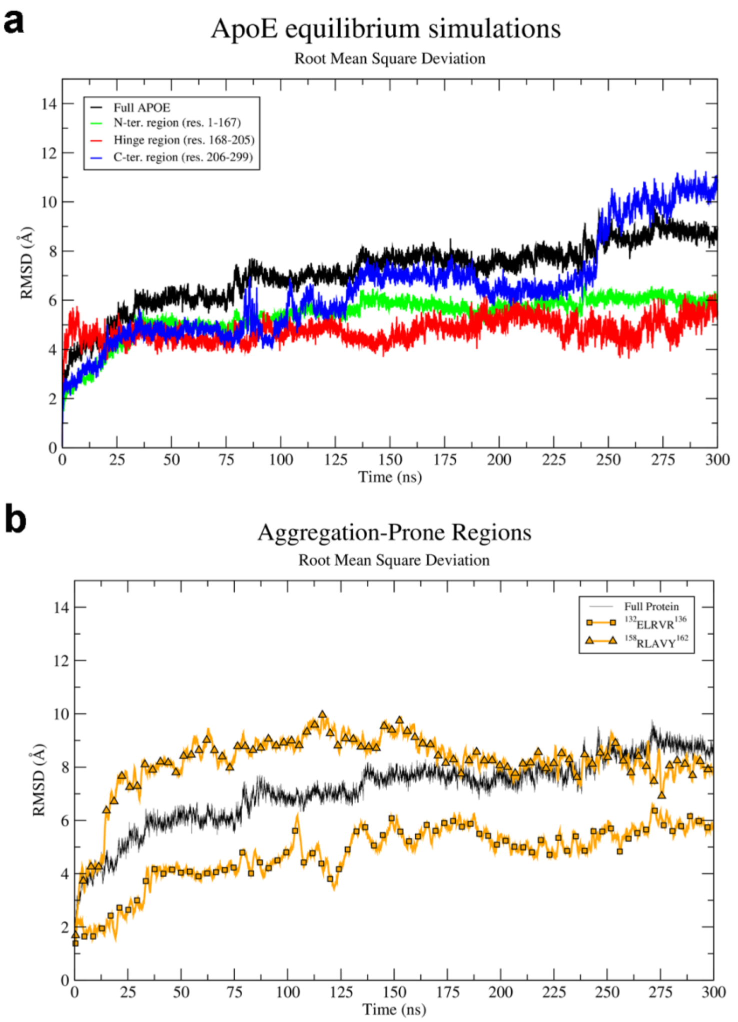

Figure S2. Molecular dynamics simulation diagrams. (a) All three domains are compared with the RMSD of the full-length apoE over time. (N-terminal domain in green, C-terminal domain in blue, hinge domain in red). **(b)** Structural deviations of ^{132}ELRVR^{136} and ^{158}RLAVY^{162} as compared with full-length apoE over 300 ns simulation time. ^{158}RLAVY^{162} exhibits 8 to 10 Å structural fluctuations.

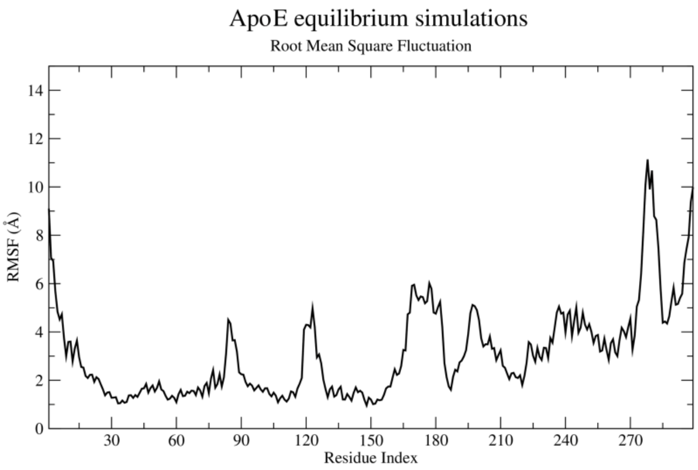

Figure S3. The average RMSF of apoE simulations. C-terminal domain exhibits the highest fluctuation rate (Please refer to the main manuscript for more details).

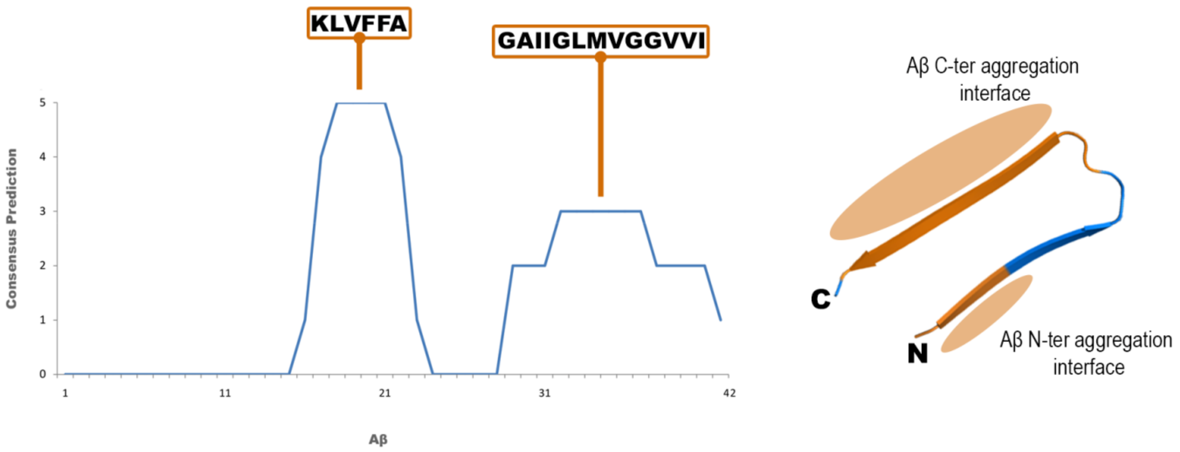

Figure S4. Amyloid propensity histograms of Aβ according to AMYLPRED [1]. Predicted Aβ “aggregation-prone” interfaces (orange) are also represented in the misfolded Aβ form obtained by 2BEG NMR structure [2].

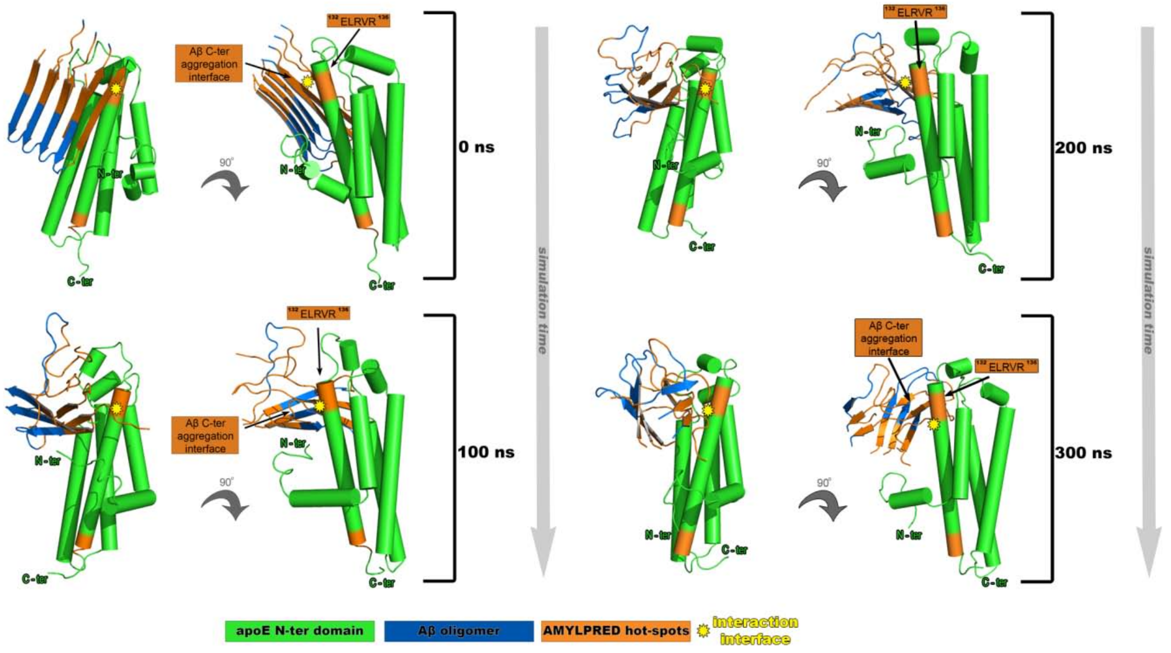

Figure S5. Molecular Dynamics simulations of Aβ-apoE complex for 300 ns. Despite the critical structural rearrangements observed for the Aβ-apoE complex over time, the C-terminal aggregation-prone

epitope of Aβ anchors the amyloidogenic ^{132}ELRVR^{136} peptide located at the N-terminal apoE domain. (N-terminal apoE domain; green, Aβ oligomer; navy blue, AMYLPRED hot-spots; orange)

References

1. Frousios, K. K.; Iconomidou, V. A.; Karletidi, C. M.; Hamodrakas, S. J., Amyloidogenic determinants are usually not buried. *BMC structural biology* **2009,** 9, 44.
2. Luhrs, T.; Ritter, C.; Adrian, M.; Riek-Loher, D.; Bohrmann, B.; Dobeli, H.; Schubert, D.; Riek, R., 3D structure of Alzheimer's amyloid-beta(1-42) fibrils. *Proc Natl Acad Sci U S A* **2005,** 102, (48), 17342-7.
